# The role of sense of coherence and loneliness in borderline personality disorder traits: a longitudinal twin study

**DOI:** 10.1186/s40479-022-00190-0

**Published:** 2022-08-01

**Authors:** Eirunn Skaug, Nikolai O. Czajkowski, Trine Waaktaar, Svenn Torgersen

**Affiliations:** 1 Department of Psychology, University of Oslo, Oslo, Norway; 2 PROMENTA Research Center, Department of Psychology, University of Oslo, Oslo, Norway; 3 Department of Mental Disorders, Norwegian Institute of Public Health, Oslo, Norway

**Keywords:** Borderline personality disorder, Sense of coherence, Loneliness, Adolescence, Longitudinal twin design

## Abstract

**Background:**

Borderline personality disorder (BPD) implies having problems with identity and relations with other people. However, not much is known about whether these indications of BPD are present in adolescence, i.e., before personality disorders usually are diagnosed. In this study, we examined the prediction of an aspect of identity (i.e., sense of coherence [SOC]) and social relations (i.e., perceived loneliness) throughout adolescence on BPD traits in young adulthood. In addition, we examined to what degree the predictive ability could be attributed to genetic and environmental factors. We also examined whether life events in adolescence were related to BPD traits.

**Methods:**

Three thousand three hundred ninety-one twins, consisting of seven national birth cohorts from Norway, participated in the study. SOC, loneliness and life events were measured three times throughout adolescence with self-report questionnaires, with 2 years in between measurements. BPD traits were measured at the end of adolescence around the age of 19 with a structured interview. Regression analyses were performed to examine the prediction of SOC, loneliness and life events on BPD traits. Cholesky decomposition models were then used to determine to what degree the associations were due to genetic and environmental influences.

**Results:**

The prediction of SOC and loneliness on BPD traits increased from R = .25 (when measured 6 years prior to the assessment of BPD traits) to R = .45 (when measured shortly before the assessment of BPD traits). In addition, negative life events considered dependent on a person’s behavior were related to BPD traits. Negative independent and positive dependent life events did not contribute to the prediction of BPD traits. Cholesky decomposition models showed that SOC and loneliness were associated with BPD traits mainly due to shared genetic influences (i.e., the proportion due to genetic influences ranged from 71 to 86%). Adding negative dependent life events to the prediction of BPD traits did not change these percentages.

**Conclusions:**

These findings indicate that the weaker SOC, the stronger feelings of loneliness, and the negative life events associated with BPD traits are mainly consequences of the genetic aspects of BPD traits, rather than having direct effects on levels of BPD symptoms.

**Supplementary Information:**

The online version contains supplementary material available at 10.1186/s40479-022-00190-0.

## Background

Borderline personality disorder (BPD) is characterized by affective instability, intensity, anger, impulsivity, and self-destructive and unstable relations to others [2]. In the alternative model for personality disorders in DSM-5, the general criteria for personality disorders includes impairment in personality functioning, defined as disturbances in self and interpersonal functioning [2]. Although these impairments are common to all personality disorders, problems related to self and others seem to be more severe in patients with BPD compared to patients diagnosed with other personality disorders [7].

Numerous studies investigating the associations between the DSM personality disorders and the Big Five personality traits have concluded that normative personality traits can be used to conceptualize personality disorders [15]. The Big Five personality traits are often found to correlate with BPD, first and foremost a profile of higher neuroticism and lower agreeableness and conscientiousness [58, 59]. Also temperamental traits in childhood, such as impulsivity, aggression [5, 18, 74, 75], high levels of emotionality, activity, shyness, and low sociability [66] are found associated with later development of BPD symptoms (e.g., affective instability, impulsivity, unstable sense of self, and interpersonal dysregulation).

Identity disturbance is a core feature of BPD [28]. However, this aspect by the disorder has received little empirical attention [25, 81]. Although identity disturbance in adolescence has been associated with number of BPD symptoms in cross-sectional studies [61, 79], knowledge about the longitudinal course of identity disturbance in the development of BPD is lacking. Identity disturbance implies problems in understanding oneself, being overwhelmed of one’s affects, lacking trust in own abilities to face challenges and finding one’s place in the world [47]. The concept of sense of coherence (SOC) is a way of looking at identity. Having a strong SOC implies perceiving stressors we face in life as clear and understandable, having confidence that one has the resources to overcome them, and finding it worthwhile to invest time and effort to cope with the situation [3]. A weak SOC, on the other hand, means perceiving oneself and the world as more chaotic, unmanageable, and meaningless. Studies examining the relationship between SOC and BPD are lacking, but several cross-sectional studies of adolescent samples have shown that SOC are associated with perceived mental and somatic health [26, 45, 55].

In addition to problems related to identity, disturbance in interpersonal functioning is another core feature of BPD. The consequence of the problems with relating to other people may be alienation, aloneness, and generally a feeling of being lonely, even when surrounded by people [31, 44]. Regarding the relationship between BPD and loneliness, only a few cross-sectional studies on BPD patients have been published [31, 40]. Examining the nature of the association between loneliness and BPD, results from a study of Australian and Dutch twins found that about half of the covariance between BPD features (i.e., affective instability, identity disturbance, negative relationships, and self-harm) and loneliness was due to shared genetic influences [60]. However, due to the cross-sectional nature of the studies, we do not know the direction of the associations, or whether the associations are time-limited related to an acute phase of BPD or have a more lasting association and potential causal effect on BPD traits. Importantly, we do not know whether these aspects of BPD are present already in adolescence before personality disorders usually are diagnosed.

BPD is associated with severe impairment in psychosocial functioning, suicidality, and the presence of comorbid mental disorders [29, 39, 64]. Although extensive research has shown that BPD usually has its onset in adolescence, diagnosing BPD in adolescence is a controversial issue, often leading to delayed diagnosis [12, 14, 43]. Furthermore, studies have shown that symptoms of BPD in adolescence are associated with long-term impairments in functioning [11, 80]. This emphasizes the importance of identifying features related to BPD in this age period. Identification of symptoms in adolescence that are associated with risk for developing BPD have important clinical implications, both in terms of prevention and early treatment [11]. Contributing to this, this paper examined whether SOC (an aspect of identity) and loneliness throughout adolescence are predictive of BPD traits in early adulthood. These constructs are closely related to how personality disorders are considered in the alternative model of personality disorders in DSM-5. In addition, impairments related to self and others are core symptoms of BPD. Both BPD [9, 54, 71], SOC [30, 62] and loneliness [27] are influenced by genetic factors. This highlights the importance of using genetically informative designs that are able to separate the environmental effect of the predictors from the potential confounding effects of shared genetic influences.

In addition to study SOC and loneliness as predictors of BPD traits, we also examined whether life events throughout adolescence influenced levels of BPD traits in early adulthood. Stressful life events represent important candidates that may influence levels of BPD traits. Childhood trauma in particular have been extensively studied, and such experiences have been implicated as important etiological risk factors in the development of BPD (e.g., [4]). Also stressful life events in adolescence have been associated with increased BPD symptoms, such as illness in the family and maladaptive family functioning [67]. In addition, studies on adult clinical samples have shown that patients with BPD report more negative recent life events compared to patients diagnosed with other personality disorders or mood disorders, and that reporting more negative life events is associated with greater impairment in functioning [50]. However, findings from genetically informative studies have challenged the commonsense interpretation of a unidirectional effect from environment to person as they have shown that environmental measures such as life events are partly influenced by genetic factors (e.g., [37]). Genetically informative studies on the relationship between environmental exposures and BPD in particular are scarce, but findings suggest that the association between BPD traits and both childhood trauma [10, 63]^1^ and life events such as divorce and job loss [20] is caused by shared genetic influences. This suggests that genes influencing BPD traits also increase the likelihood of being exposed to childhood trauma and certain life events. Clearly, more genetically informative studies are needed to enhance our understanding of the relationship between assumed environmental risk factors and BPD.

The research objectives presented above can be specified into five aims. The first aim was to examine to what extent BPD traits in early adulthood can be predicted from SOC and loneliness in adolescence. The second aim was to examine whether life events in adolescence are predictive of BPD symptoms, separately and together with SOC and loneliness. The third aim was to determine to what degree associations between SOC, loneliness and BPD traits can be attributed shared genetic and environmental influences. The fourth aim, building on the third aim, was to examine whether accounting for life events in adolescence changes the estimated contribution of genetic and environmental influences. Finally, the fifth aim was to look at the development of SOC and loneliness throughout adolescence into the post-adolescence years as one kind of longtime borderline trait, and estimate the relative contribution of genetic and environmental influences of a common factor consisting of SOC, loneliness and BPD.

## Method

### Participants

Data for the study were drawn from the Oslo University Adolescent and Young Adult Twin Project [72, 73]. All twin pairs born in Norway between 1988 and 1994 were invited to participate. The twins completed self-report questionnaires three times throughout adolescence, with 2 years in between (12 to 18 years at Wave-1). In addition, the twins participated in a face-to-face interview when they were around age 19 (*M* = 19.1, *SD* = 1.2). Informed consent was obtained from both the twins and their parents. The project was approved by the Norwegian Data Inspectorate and the Regional Committees for Medical and Health Research Ethics. American Psychological Association ethical standards were followed in the conduct of the study.

In the present study, we rearranged the self-report questionnaire data from Wave-1, Wave-2, and Wave-3 data (in which each wave included data from seven birth cohorts) into data from the age of 12–13 years, 14–15 years, 16–17 years, and 18 years and older (i.e., until the time of the interview assessment of BPD traits). The whole sample consisted of 3391 twins (56% females) from 1716 twin pairs. All twins, from both complete and incomplete pairs, were included in the study. Table 1 displays sample characteristics derived from the questionnaire data and the interview data. The majority of those who responded to questionnaires, also participated in the interview (i.e., 76, 80, 82 and 87% at age 12–13, 14–15, 16–17 and 18, respectively).

**Table 1:** Twin sample characteristics

	N single twins	N twin pairs	MZ twin pairs^a^	DZ twin pairs^a^
Questionnaire-data				
12–13 years	852	432	165	255
14–15 years	1501	767	276	458
16–17 years	1792	922	329	541
18 years	1371	782	221	368
Interview-data	2808	1424	541	843

*MZ* monozygotic, *DZ* dizygotic

^a^Number of complete pairs

### Zygosity determination

The zygosity of same-sex twin pairs were partially determined through a 12-item zygosity scale where questions about similarity in appearance, how often the twins have been mixed-up with each other, and whether they believe that they are monozygotic or dizygotic were asked [68]. To validate the zygosity scale, cheek swabbed DNA was drawn from 513 of the 1006 same-sex twin pairs. Seventeen genetic markers were tested, with an estimated probability of misclassification less than *p* < 0.0001. The scores on the zygosity scale were analyzed using discriminant analysis and a cutting point for the discriminant score was established based on the results of the gene testing. Those with a discriminant score close to the cutting point were oversampled for DNA tests. It appeared that 14 out of the 513 twin pairs were misclassified according to the discriminant analysis. Correcting for the oversampling, the questionnaire misclassified 2.13% of the same-sex twins. However, as almost all of the misclassified pairs were gene tested, only 0.64% of the same-sex twin pairs are expected to be misclassified (0.45% when including the whole twin sample).

### Measures

#### Questionnaire data: sense of coherence, loneliness and life events

SOC was measured by an abbreviated 5-item version of the Sense of Coherence 13-item scale (SOC-13 [3];). The abbreviation of the SOC-13 scale was performed based on results from a pilot study [72]. The SOC-13 scale has been shown to have good internal consistency, with Cronbach’s alpha ranging from 0.70 to 0.92 across studies [22]. The Cronbach’s alpha of the SOC scale used in this study ranged from 0.82 to 0.83 across the study waves, supporting the reliability of the abbreviated 5-item scale. The scale included the following questions: “Do you have the feeling that you are being treated unfairly?”, “Do you have the feeling that you are in an unfamiliar situation and don’t know what to do?”, “Do you have very mixed-up feeling and ideas?”, “Does it happen that you have feelings inside you would rather not feel?” and “How often do you have the feeling that there’s little meaning in the things you do in your daily life?”. Reponses were given on a 7-point Likert scale ranging from 1 (*very often*) to 7 (*rarely/never*). Average scores were computed with higher scores indicating stronger SOC.

Loneliness was measured by a 5-item scale, including a 4-item survey version of the R-UCLA Loneliness scale ([56]; “I feel in tune with the people around me”, “I can find companionship when I want it”, “No one really knows me well”, “People are around me but not with me”) and one direct measure of loneliness (“I feel lonely”). Responses were given on a 5-point Likert scale ranging from 0 (*not typical*) to 4 (*very typical*). Positively worded items were reverse-coded, and average scores were computed with higher scores indicating higher levels of loneliness. There were strong correlations between the single direct measure of loneliness and the aggregate of the four R-UCLA items across all age groups (i.e., the correlations ranged from *r* = .60 to *r* = .68). Furthermore, the Cronbach’s alpha of the full loneliness scale ranged from 0.77 to 0.84 across the study waves.

Life events were measured by a 38-item scale asking whether the participants had experienced any of the set of life events the past year (0 = *no*; 1 = *yes*). Twenty-nine events came from the Life Event Questionnaire for Adolescents (LEQ-A [42];) and nine events were added to the scale after a pilot study (see Table S1). The life events were divided into three clusters; negative life events considered dependent on a person’s behavior (e.g., “I had many arguments with my parents”), negative life events considered independent on a person’s behavior (e.g., “One of my parents died”) and positive life events considered dependent on a person’s behavior (e.g., “I got a new friend”). Life events from the LEQ-A were assigned to clusters according to Masten et al. [42], and the remaining events were classified based on the authors’ evaluation. Sum scores of the respective life events clusters were used when analyzing the data, with possible values ranging from 0 to 14 (negative dependent), 0–19 (negative independent) and 0–5 (positive dependent).

#### Interview data: borderline personality disorder traits

A Norwegian version of the Structured Interview for DSM-IV Personality (SIDP-IV [51];) was used to assess BPD traits [32]. Each twin in a pair was interviewed by different interviewers. The SIDP-IV uses a five-year rule, which means that the ratings are based on behavior typical for the past 5 years. Each criterion is scored on a 4-point scale from 0 to 3 (0 = *absent*; 1 = *subthreshold*; 2 = *present*; 3 = *strongly present*). At least five of nine criteria are required for a BPD diagnosis. The prevalence for a categorically defined BPD diagnosis in the present sample was too low to perform reliable analyses. We therefore studied BPD as a dimensional trait by calculating the number of endorsed criteria either at the clinical or subclinical level (≥ 1). Interrater reliability was assessed based on two raters’ scoring of 55 audiotaped interviews, of which 53 of the recordings were of satisfactory quality to be scored. The intraclass correlation coefficient for the dimensional measure of BPD (hereafter referred to as BPD traits) was 0.77 (*p* < 0.001).

### Statistical analyses

All analyses were performed in the statistical package R [53]. First, we assessed the phenotypic associations between SOC, loneliness and BPD traits using correlation and linear regression analyses. Four regression analyses were performed, each with BPD traits as the dependent variable, and SOC and loneliness at a given age as independent variables (i.e., 12–13 years, 14–15 years, 16–17 years or 18 years). We then examined whether life events contributed to the prediction of BPD by adding life events (negative dependent, negative independent and positive dependent) to the regression analyses.

Next, the classical twin design was used to partition the phenotypic correlations between the predicted scores (derived from the regression analyses) and BPD traits into genetic and environmental influences. Twin models allow the variance of an observed phenotype (and the covariance between phenotypes) to be partitioned into three sources; additive genetic (A), shared environmental (C) and non-shared environmental (E) factors. The classical twin design relays on comparing the correlation within monozygotic (MZ) pairs with the correlation within dizygotic (DZ) pairs. MZ twins are genetically identical whereas DZ twins share, on average, half of their segregating genes. Thus, influence of A is inferred when the MZ correlation exceeds the DZ correlation. Furthermore, both MZ and DZ twins experience environments that are shared by both twins within a pair. If these experiences contribute to phenotypic similarity within pairs, they are attributed influence of C. Influence of C is inferred when the DZ correlation is more than half the magnitude of the MZ correlation. Finally, the E effects represent all experiences that contribute to phenotypic dissimilarity within pairs, including measurement error.

In the same way, bivariate twin models allow us to partition the covariance between phenotypes into genetic and environmental influences. More specifically, we fitted a series of bivariate Cholesky decomposition models to quantify how much of the phenotypic correlations between the predicted scores and BPD traits that were due to genetic and environmental factors, respectively. The predicted scores from a given age was included as the first variable, with BPD traits as the second variable in each model. Using data from twins, the bivariate Cholesky decomposition partitions the variation in the first variable into genetic and environmental sources and quantify the extent in which those genetic and environmental sources also contribute to the variance in the second variable. The remaining variance in the second variable that is not shared with the first variable is also partitioned into genetic and environmental sources [49]. The analyses were conducted in the structural equation modeling package OpenMx [48]. Models were fitted to raw data using full information maximum likelihood. We first fitted full ACE models, followed by reduced models. Model fit was evaluated based on the models Akaike’s information criterion (AIC), with lower values indicating better model fit [1]. For each age group, we report the proportion of the phenotypic correlation between the predicted scores and BPD traits that was due to genetic and environmental factors, respectively.

Finally, we created a factor including the measures of SOC and loneliness throughout adolescence and BPD traits in young adulthood. Missing data were imputed using multiple imputation by fully conditional specification [76]. The mean factor score based on the scores from 10 iterations were computed and then used in a univariate twin model to determine the heritability of this ‘longtime borderline trait’.

## Results

### Descriptive statistics and phenotypic associations

Table 2 display descriptive statistics for each study variable from the questionnaire data. For BPD traits, measured with a diagnostic interview around the age of 19 (*M* = 19.1, SD = 1.2), the mean was 1.08 (*SD* = 1.60). Inter-scale correlations and correlations with sex are provided in Table S2. Overall, the correlations between sex and all study variables were weak, ranging from *r* = −.01 to *r* = −.22.

**Table 2:** Descriptive statistics for study variables

Variable	12–13 years	14–15 years	16–17 years	18 years
	*M (SD)*	*M (SD)*	*M (SD)*	*M (SD)*
SOC	5.42 (1.21)	5.09 (1.23)	4.88 (1.30)	4.95 (1.28)
LON	1.00 (0.68)	1.05 (0.71)	1.08 (0.75)	1.07 (0.76)
NegDep	2.01 (1.89)	2.50 (2.13)	2.48 (2.08)	2.35 (2.03)
NegInd	1.41 (1.26)	1.20 (1.31)	1.69 (1.46)	1.46 (1.43)
PosDep	2.09 (1.30)	2.04 (1.28)	2.45 (1.17)	1.99 (1.24)

*SOC* sense of coherence, *LON* loneliness, *NegDep* negative dependent life events, *NegInd* negative independent life events, *PosDep* positive dependent life events

Table 3 presents correlations and results from linear regression analyses predicting BPD traits in early adulthood from SOC and loneliness at four different ages throughout adolescence. SOC were negatively associated with BPD traits, whereas loneliness showed positive associations with BPD traits. As expected, the strength of the associations increased as the time-lag between measures of SOC, loneliness and BPD traits decreased.

**Table 3:** Pearson correlations and results from linear regression analyses, predicting BPD traits from SOC and loneliness

Age group	Pearson correlation with BPD traits		Standardized Beta		R
	SOC	LON	SOC	LON	
12–13 years	−.21^***^	.20^***^	−0.16^***^	0.14^***^	.25
14–15 years	−.28^***^	.27^***^	−0.21^***^	0.18^***^	.33
16–17 years	−.33^***^	.29^***^	−0.24^***^	0.19^***^	.37
18 years	−.44^***^	.30^***^	−0.38^***^	0.12^***^	.45

*BPD traits* borderline personality disorder traits, *SOC* sense of coherence, *LON* loneliness, *R* coefficient of multiple correlation

^***^p < 0.001

Table 4 presents correlations between life events and BPD traits. Negative dependent and negative independent life events showed weak positive associations with BPD traits, whereas the associations between positive dependent life events and number of BPD symptoms were negligible.

**Table 4:** Pearson correlations between BPD traits and life events throughout adolescence

Age group	Pearson correlation with BPD traits		
	Negative dependent life events	Negative independent life events	Positive dependent life events
12–13 years	.24^***^	.06	.07
14–15 years	.28^***^	.17^***^	.04
16–17 years	.34^***^	.14^***^	.07^**^
18 years	.35^***^	.17^***^	.01

*BPD traits* borderline personality disorder traits

^**^p < 0.01

^***^p < 0.001

When life events were included in the regression analyses (see Table 5), the coefficient of multiple correlation (R) slightly increased compared to the models predicting BPD traits from SOC and loneliness, only. Of note, it was negative dependent life events that contributed to the prediction of BPD traits. Although negative independent life events showed weak bivariate correlations with BPD traits, this cluster of life events did not have any independent effect on number of BPD symptoms.

**Table 5:** Results from linear regression analyses, predicting BPD traits from SOC, loneliness and life events

Age group	Standardized Beta					R
	SOC	LON	NegDep	NegInd	PosDep	
12–13 years	−0.09	0.13^**^	0.18^***^	−0.02	0.00	.30
14–15 years	−0.11^**^	0.16^***^	0.16^***^	0.07^*^	−0.01	.36
16–17 years	−0.13^***^	0.20^***^	0.22^***^	0.00	0.03	.42
18 years	−0.29^***^	0.12^***^	0.18^***^	0.02	−0.04	.48

*BPD traits* borderline personality disorder traits, *SOC* sense of coherence, *LON* loneliness, *NegDep* negative dependent life events, *NegInd* negative independent life events, *PosDep* positive dependent life events, *R* coefficient of multiple correlation

^*^p < 0.05

^**^p < 0.01

^***^p < 0.001

Using data from twins allow us to examine to what degree SOC and loneliness predicts BPD traits because these phenotypes share genetic influences, and to what degree the associations are due to environmental influences contributing to variation in both the predictor variables and BPD traits. More specifically, we fitted a series of bivariate Cholesky decompositions to partition the phenotypic correlations between the predicted scores derived from the regression analyses and BPD traits into genetic and environmental influences. Table 6 presents cross-trait correlations between the predicted scores (i.e., derived from the regression analyses with SOC and loneliness as independent variables) and BPD traits. The pattern of twin correlations suggests that the associations between SOC, loneliness and BPD traits are mainly due to genetic influences, with no influence of shared environmental factors (i.e., the DZ correlations were not greater than half the size of the MZ correlations). Furthermore, the slightly lower MZ correlations compared to the phenotypic correlations suggest small non-shared environmental influences between SOC, loneliness and BPD traits. Overall, the same pattern of twin correlations was observed when negative dependent life events were added to the predicted scores (see Table S3).

**Table 6:** Cross-trait correlations

Variable^a^	Correlation with BPD traits		
	Phenotypic [95% CI]	rMZ [95% CI]	rDZ [95% CI]
SOC and LON 12–13 years	.25 [.18, .32]	.21 [.09, .33]	.10 [.00, .20]
SOC and LON 14–15 years	.33 [.27, .38]	.26 [.18, .35]	.09 [.02, .16]
SOC and LON 16–17 years	.37 [.33, .41]	.33 [.26, .40]	.11 [.05, .18]
SOC and LON 18 years	.45 [.40, .50]	.38 [.29, .45]	.19 [.12, .26]

*BPD traits* borderline personality disorder traits, *SOC* sense of coherence, *LON* loneliness, *Phenotypic* correlation without considering twin-pair membership, *rMZ* cross-twin correlation between monozygotic twin pairs, *rDZ* cross-twin correlation between dizygotic twin pairs

^a^Predicted scores for BPD traits derived from linear regression analyses, with SOC and LON at different ages as independent variables

**Fig. 1:**
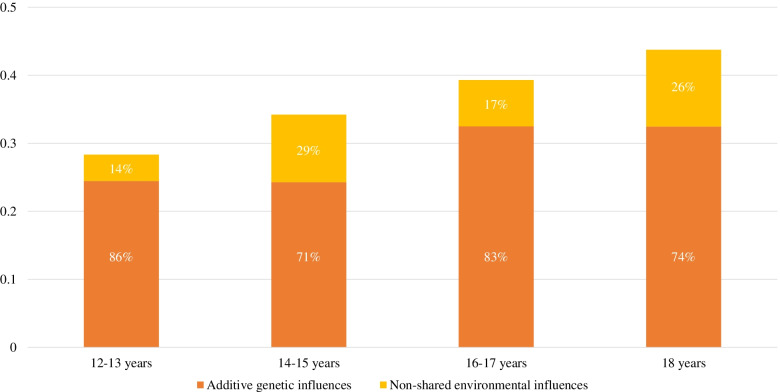
Genetic and Environmental Influences on the Association between the Predicted Scores and BPD Traits. *Note.* BPD traits = borderline personality disorder traits; Predicted scores = predicted scores for BPD traits derived from linear regression analyses, with sense of coherence and loneliness at different ages as independent variables. The height of the bars represents the phenotypic correlation between the predicted scores and BPD traits. The percentages represent the proportions of the phenotypic correlations due to genetic and environmental influences

According to the AIC values, an AE model (i.e., dropping the shared environmental parameters) was the best fitting model for all Cholesky decomposition models. This is also consistent with results from univariate twin analyses, where an AE model was found to have best fit for all predictor variables. All variables were moderately heritable (see Table S4). The proportions of the phenotypic correlations between the predicted scores (i.e., based on SOC and loneliness) and BPD traits due to genetic and environmental influences are displayed in Fig. 1 as a set of stacked bar charts. For standardized parameter estimates derived from the Cholesky decomposition models, see Table S5. The results indicated that the phenotypic correlations between the predicted scores and BPD traits were mainly due to additive genetic influences, with additive genetic influences accounting for between 71 and 86% of the phenotypic correlations. When negative dependent life events were added to the predicted scores, the relative contribution of genetic and environmental influences were close to identical (in fact slightly higher contributions of genetic influences) to the proportions displayed in Fig. 1 (i.e., the proportion of the phenotypic correlations due to additive genetic influences were 89, 73, 85 and 74% at age 12–13, 14–15, 16–17 and 18, respectively). See Fig. S1 for a figurative illustration of the results and Table S5 for standardized parameter estimates. Genetic and environmental correlations provided support for the results provided in Fig. 1. That is, the genetic correlations between the predicted scores and BPD traits were moderate to high, whereas the environmental correlations were weak, around 1/4 of the genetic correlations (see Table S6).

Finally, we created a factor of all measures of SOC and loneliness throughout adolescence, and BPD traits (for factor loadings, see Table S7). In this way, we looked at SOC and loneliness as a kind of longtime borderline trait. Univariate twin analyses showed that the heritability of this factor was .56, 95% CI [.52, .61], which is somewhat higher compared to the heritability of BPD traits measured at one time point (h^2^ = .50, 95% CI [.44, .55]).

## Discussion

The present study examined if SOC and loneliness in adolescence can predict BPD traits in early adulthood. Conceptually, these possible predictors of BPD traits are closely related to disturbances in self- and interpersonal functioning, which is how the alternative model for personality disorders in DSM-5 characterizes personality disorders [2]. Furthermore, previous research has shown that BPD usually has its onset in adolescence and have highlighted the importance of early detection and intervention to prevent chronicity and reduce the risk for long-term consequences [14, 35, 43]. The present study demonstrated that SOC and loneliness already at the age of 12 is predictive of BPD traits in early adulthood (*M* = 19.1 years). The correlation between the predicted and the observed BPD scores increased as the time interval between the measurements decreased, and the twins got older. We cannot know if the prediction increased in strength due to decreased time interval between measurements or due to an effect of age. Previous studies have shown that both SOC [22, 34] and loneliness [46] are relatively stable constructs across the life span, showing similar rank-order stability as personality traits [17]. Thus, it is reasonable to believe that SOC and loneliness measured at earlier ages are precursors of later measures of SOC and loneliness.

Regarding the association between loneliness and BPD traits, a few cross-sectional studies exist, all finding positive associations between loneliness and BPD [31, 40, 60]. Studies examining the relationship between SOC and BPD are lacking, but previous studies have described associations between BPD traits and features related to SOC such as poor functioning in response to stress [5], lack of effective emotion regulation strategies and difficulties with goal-directed behavior [57]. A weak SOC means, at the extreme, perceiving oneself and the world as chaotic, unmanageable, and meaningless. Hence, SOC may relate to the identity disturbance associated with BPD, with difficulties finding meaning in life, difficulties with self-direction and feelings of worthlessness [25, 47, 61, 79]. The associations between SOC, loneliness and BPD traits may also be related to personality traits. For example, lonely people are shown to be characterized by a profile of higher neuroticism and lower extraversion compared to less lonely people [13]. SOC has also been associated with the Big Five traits, especially neuroticism [24, 33]. A research objective for future studies may therefore be to examine whether personality traits can explain some of the predictive power of SOC and loneliness on BPD traits.

The present study utilized a longitudinal twin design, allowing sequencing of predictors and outcome, and examination of the nature of the association between the predictor variables and BPD traits. The results showed that SOC and loneliness are associated with BPD traits mainly for genetic reasons, and that the relative contribution of genetic and environmental influences was more or less the same throughout adolescence. More specifically, although the associations between SOC, loneliness and BPD traits increased as the time-lag between the assessments decreased, the proportion of the correlations due to genetic and environmental influences remained quite stable (i.e., proportions due to genetic influences varied from 71 to 86%). The results showed moderate to high genetic correlations and weak environmental correlations between the phenotypes, providing further support to the observation that the associations were mainly due to genetic influences. In sum, the results indicate that the genetic factors that influence BPD symptoms also increase the likelihood of having a weaker SOC and stronger feelings of loneliness. To our knowledge, only one previous study has examined the nature of the association between loneliness and BPD. This study found that 51% of the phenotypic correlation between loneliness and BPD traits was due to genetic influences [60]. Results of the present study showed a higher proportion due to genetic influences. However, we studied the combined effect of SOC and loneliness on BPD traits, and thus our results are not directly comparable with the study by Schermer et al. [60].

Although most of the phenotypic correlations were due to shared genetic influences, the results also indicate that common genes are not the only explanation for the association between SOC, loneliness and BPD traits. Between 14 and 29% of the correlations were due to shared non-shared environmental influences, possibly indicating that changing the environmental factors that affect a person’s SOC or loneliness may also influence symptoms of BPD.

Negative dependent and negative independent life events were also associated with BPD traits, but positive dependent life events were not (with a small exception for those in the 16-17 age group). When a multiple regression analysis was conducted, only negative dependent life events survived together with SOC and loneliness. Adding negative dependent life events to the prediction of BPD traits did not change the proportions of the phenotypic correlations due to additive genetic and environmental influences. It is noteworthy that although ‘measured environments’ such as negative dependent life events predict BPD, they do not add anything to the relative contribution of environmental influences. That is, measures of the environment are not always ‘environmental’, as genetically informative studies have shown (e.g., [37]). Together, the results suggest that SOC, loneliness and negative dependent life events throughout adolescence are associated with BPD traits in early adulthood mainly due to shared genetic influences. Analyses of the genetic and environmental contribution to stability in the measured constructs further support these results, as they showed that the stability of both SOC, loneliness and the recurrence of life events were mainly due to genetic influences.

Finally, a factor of SOC and loneliness throughout adolescence, and BPD traits in early adulthood showed a heritability of 56%, which is somewhat higher than looking at BPD traits alone. The heritability of this factor is also higher than the usual 40-50% found when studying personality traits and personality disorders on a specific occasion [41, 69, 78], and is probably a better approximation of the real contribution of genetic influences on BPD traits. Assessing a personality trait or personality disorder at a specific occasion means a lot of time-specific influence, chance variance and measurement error. Furthermore, this time-specific measurement error cannot be separated from the estimate of non-shared environmental influences, leading to an overestimation of the non-shared environmental influences and a corresponding underestimation of genetic influences. For example, in a full-population study of individuals born in Sweden between 1973 and 1993, the heritability of clinically diagnosed BPD was estimated to 46% [65]. Similar estimates have been reported in other cross-sectional studies that have used dimensional measures of BPD traits [10, 21, 36, 70]. Longitudinal studies, on the other hand, have reported heritability estimates of BPD traits up to 70% [9, 54]. In general, any personality trait or disorder should be assessed in a longer life perspective as they are by definition relatively long-lasting, and retrospective reports are usually highly unreliable and are missing important information.

### Limitations and strengths

The results should be considered in light of several possible limitations. First, our measure of BPD traits includes both subclinical and clinical scores and therefore may not generalize to clinical populations. Second, although the global measure of loneliness used in the present study are frequently used in the research literature, subtypes of loneliness may have differential associations with BPD traits. For example, results from a study by Lasgaard, Goossens, Bramsen, Trillingsgaard, and Elklit [38] indicated that peer-related and family-related loneliness showed differential associations with psychopathology in adolescence. Future studies may examine the effect of multiple dimensions of loneliness on BPD traits. Third, although it is common to categorize life events based on event-dependence, some events may not be clearly classified as independent or dependent (e.g., event-dependence for some events such as ‘moving schools’ may vary across individuals). A clear criterion for classification of event-dependence is difficult to obtain without knowing the ‘causes’ behind the experiences (e.g., was the person having many arguments with his/her sibling due to own behavior or was it due to the sibling’s quarrelsome behavior?). However, prior studies have reported higher heritability of dependent life events compared to independent life events, supporting a differentiation between them [6, 8, 52]. Studying classes of life events may provide a more accurate picture of the effect of life events on various outcomes. Results from the present paper supports a division of life events into different clusters as their effect on BPD traits strongly differed. Fourth, all measures are based on self-reports from the twins. Both loneliness and SOC are intended to measure a person’s subjective feelings and perceptions of the world, making self-reports the most appropriate approach. However, it is possible that the measure of BPD traits could have been improved (i.e., more reliable) by reports from significant others in addition to the twins’ own reports. Whether the use of self-reports represent a limitation or not may depend on the person being asked. For example, although self-reports may be prone to subjective interpretations, the responses mimic the clinical situation where the clinician has to rely on the patient’s descriptions. Fifth, some of the measures were markedly skewed. When fitting structural equation models to non-normal data, this could result in underestimated standard errors. Thus, individual parameters may be statistically significant more frequently than they should be. Sixth, there are several assumptions related to the classical twin design which may threat the validity of results if they are violated. Violations of the equal environment assumption (EEA) that MZ and DZ twin pairs experience the same degree of environmental similarity may result in overestimation of the effect of genetic influences [23]. However, empirical evidence supports the validity of the EEA (e.g., [16, 19]). Another major assumption is that DZ twin pairs share half of their segregating genes. This is based on the assumption of random mating. If mating is not random, parents may share genes beyond what is expected by chance. Consequently, DZ twin pairs will share more than 50% of their segregating genes. If the phenotypes under study have been subject to non-random mating (i.e., assortative mating), findings will overestimate the shared environmental influences and underestimate the genetic influences. Positive assortative mating would also lead to an overestimation of genetic correlations [77]. However, previous studies have found assortative mating to be low for related phenotypes such as personality domains [49].

The study also has several strengths. The present study extends previous research by using a longitudinal design which allow sequencing of predictor and outcome. Furthermore, using data from twins makes it possible to partition the covariance between phenotypes into genetic and environmental influences. In this way, one can determine to what degree a predictor is associated with BPD traits due to a direct ‘environmental’ effect of the predictor and to what degree the phenotypes are associated due to shared genetic influences. Furthermore, data for the study consisted of a full cohort population-based sample, which strengthens the possibility of generalization of findings. However, future studies should seek to replicate findings in different samples from other countries.

## Conclusion

SOC and loneliness already at age 12 years is associated with increased levels of BPD symptoms in young adulthood. The associations increased in strength with older age and shorter time until assessment of BPD traits. Negative dependent life events were also associated with BPD traits partly independent of the effects of SOC and loneliness. Results from the present study suggest that SOC, loneliness and negative dependent life events are associated with BPD mainly because they share common genetic influences, rather than a direct/causal effect of the predictors on levels of BPD symptoms. That is, the predictors seem to be consequences of the genetic aspects related to BPD.

## Supplementary Information

**Additional file 1: Table S1.** The Life Events Scale. **Table S2.** Inter-Scale Correlations. **Table S3.** Cross-trait Correlations. **Table S4.** Univariate Model Estimates From the Best Fitting Twin Models. **Table S5.** Standardized Parameter Estimates from the Bivariate Cholesky Decomposition Models. **Table S6.** Genetic and Environmental Correlations Derived from the Bivariate Cholesky Decomposition Models. **Table S7.** Factor Loadings. **Figure S1.** Genetic and Environmental Influences on the Association between the Predicted Scores and BPD Traits.

## Data Availability

The dataset analyzed during the current study are not publicly available. Data collection for the study was preapproved in 2005 by the Norwegian Data Protection Authority (DPA) under a clause of 20 years individual data protection and subsequent data deletion or anonymization. Anonymized data may be requested after 2025.

## Abbreviations

AIC: Akaike’s information criterion
BPD traits: Borderline personality disorder traits
LEQ-A: Life Event Questionnaire for Adolescents
SIDP-IV: Structured Interview for DSM-IV Personality
SOC: Sense of coherence
A: Additive genetic factors
C: Shared environmental factors
E: Non-shared environmental factors
MZ: Monozygotic
DZ: Dizygotic

## Footnotes

1 The study by Skaug et al. used data from the same dataset as the present study.
