# Supplementary material for: The role of sense of coherence and loneliness in borderline personality disorder traits: a longitudinal twin study
Source: Borderline Personal Disord Emot Dysregul. 2022 Aug 1;9:19. doi: 10.1186/s40479-022-00190-0 (PMC9341038; doi:10.1186/s40479-022-00190-0)
Supplement: Supplementary file 1 — Additional file 1: Table S1. The Life Events Scale. Table S2. Inter-Scale Correlations. Table S3. Cross-trait Correlations. Table S4. Univariate Model Estimates From the Best Fitting Twin Models. Table S5. Standardized Parameter Estimates from the Bivariate Cholesky Decomposition Models. Table S6. Genetic and Environmental Correlations Derived from the Bivariate Cholesky Decomposition Models. Table S7. Factor Loadings. Figure S1. Genetic and Environmental Influences on the Association between the Predicted Scores and BPD Traits. [file 40479_2022_190_MOESM1_ESM.docx]

Supplementary Material

**Table S1**

*The Life Events Scale*

| **Negative dependent life events** |
| --- |
| I had an important change in physical appearance, which upset me (acne, glasses, physical development, etc.)^a^ |
| I was a victim of violence (mugging, sexual abuse, robbery)^a^ |
| I was disappointed by a friend |
| I was disappointed by someone in the family |
| I did not get into a group or activity that I wanted to get into (music group, sports team, theater, etc.)^a^ |
| I had major problems with a teacher |
| I did much worse than I expected in an important exam or course^a^ |
| I had less contact with one of my parents^a^ |
| I had many arguments with my siblings^a^ |
| I had many arguments with my parents^a^ |
| I was bullied by other pupils/adolescents |
| I broke up with a girlfriend/boyfriend^a^ |
| I had an abortion (girls) / my girlfriend had an abortion (boys) |
| I lost a close friend^a^ |
| **Negative independent life events** |
| I lost a pet |
| I changed schools^a^ |
| I became seriously ill or was injured^a^ |
| At least one parent or another family member became seriously ill or was injured^a^ |
| One of my parents died^a^ |
| A brother or sister died^a^ |
| Another family member died^a^ |
| One of my close friends died^a^ |
| Mom or Dad’s friend moved in with us^a^ |
| A member of my family ran away from home^a^ |
| My parents divorced, moved apart^a^ |
| One of my parents had problems at work^a^ |
| One parent lost his or her job^a^ |
| My mother began to work^a^ |
| There has been a change in a parent’s job so that my parent is away from home more often^a^ |
| The family financial situation was difficult^a^ |
| There was some damage or loss of family property (such as apartment, house, car or bike)^a^ |
| There were many arguments between the adults^a^ |
| Someone in the family had problems with the police^a^ |
| **Positive dependent life events** |
| I received a special award (trophy, diploma etc.) for something done at school^a^ |
| I became more popular with my friends |
| I joined a fun group of friends |
| I got a boyfriend/girlfriend^a^ |
| I got a new friend |

*Note.* ^a^ Question from the Life Event Questionnaire for Adolescents (LEQ-A; Masten et al., 1994). The wording in some of the questions were slightly changed from the LEQ-A.

**Table S2**

*Inter-Scale Correlations*

|  | Variable | Sex ^a^ | SOC | LON | NegDep | NegInd |
| --- | --- | --- | --- | --- | --- | --- |
| 12–13 years | |  |  |  |  |  |
|  | SOC | -.09^**^ | – |  |  |  |
|  | LON | .06 | -.37^***^ | – |  |  |
|  | NegDep | .13^***^ | -.48^***^ | .22^***^ | – |  |
|  | NegInd | -.01 | -.17^***^ | .10^**^ | .30^***^ | – |
|  | PosDep | .06 | -.19^***^ | -.11^**^ | .32^***^ | .21^***^ |
| 14–15 years | |  |  |  |  |  |
|  | SOC | -.20^***^ | – |  |  |  |
|  | LON | .04 | -.42^***^ | – |  |  |
|  | NegDep | .22^***^ | -.54^***^ | .24^***^ | – |  |
|  | NegInd | .09^***^ | -.29^***^ | .13^***^ | .40^***^ | – |
|  | PosDep | .07^**^ | -.15^***^ | -.17^***^ | .32^***^ | .17^***^ |
| 16–17 years | |  |  |  |  |  |
|  | SOC | -.22^***^ | – |  |  |  |
|  | LON | .06^**^ | -.44^***^ | – |  |  |
|  | NegDep | .22^***^ | -.49^***^ | .22^***^ | – |  |
|  | NegInd | .10^***^ | -.28^***^ | .10^***^ | .39^***^ | – |
|  | PosDep | .07^**^ | -.13^***^ | -.17^***^ | .30^***^ | .19^***^ |
| 18 years | |  |  |  |  |  |
|  | SOC | -.17^***^ |  |  |  |  |
|  | LON | .03 | -.48^***^ | – |  |  |
|  | NegDep | .20^***^ | -.49^***^ | .22^***^ | – |  |
|  | NegInd | .10^***^ | -.26^***^ | .13^***^ | .42^***^ | – |
|  | PosDep | .02 | -.05^*^ | -.14^***^ | .26^***^ | .18^***^ |

*Note.* SOC = sense of coherence; LON = loneliness; NegDep = negative dependent life events; NegInd = negative independent life events; PosDep = positive dependent life events. Correlations with BPD traits are provided in Table 3 and Table 4. ^a^ Sex coded 0 = male, 1 = female. The correlation between sex and BPD traits was *r* = .11^***^.

^**^*p* < 0.01. ^***^*p* < 0.001.

**Table S3**

*Cross-trait Correlations*

|  | | Correlation with BPD traits | | |
| --- | --- | --- | --- | --- |
| Variable ^a^ | | Phenotypic [95% CI] | rMZ [95% CI] | rDZ [95% CI] |
| SOC, LON and NegDep | |  |  |  |
|  | 12–13 years | .30 [.22, .37] | .23 [.10, .34] | .18 [.08, .27] |
|  | 14–15 years | .36 [.31, .41] | .27 [.18, .35] | .15 [.08, .22] |
|  | 16–17 years | .42 [.38, .46] | .37 [.29, .44] | .18 [.11, .24] |
|  | 18 years | .48 [.43, .52] | .38 [.30, .46] | .23 [.16, .30] |

*Note.* BPD traits = borderline personality disorder traits; SOC = sense of coherence; LON = loneliness; NegDep = negative dependent life events; Phenotypic = correlation without considering twin-pair membership; rMZ = cross-twin correlation between monozygotic twin pairs; rDZ = cross-twin correlation between dizygotic twin pairs. ^a^ Predicted scores for BPD traits derived from linear regression analyses, with SOC, LON and NegDep at different ages as independent variables.

**Table S4**

*Univariate Model Estimates From the Best Fitting Twin Models*

|  | | Additive genetic effects | Non-shared environmental effects |
| --- | --- | --- | --- |
| 12–13 years | |  |  |
|  | SOC | .40 [.28, .50] | .60 [.50, .72] |
|  | LON | .45 [.34, .55] | .55 [.45, .66] |
|  | NegDep | .60 [.51, .67] | .40 [.33, .49] |
| 14–15 years | |  |  |
|  | SOC | .46 [.37, .54] | .54 [.46, .63] |
|  | LON | .49 [.41, .56] | .51 [.44, .59] |
|  | NegDep | .54 [.47, .61] | .46 [.39, .53] |
| 16–17 years | |  |  |
|  | SOC | .38 [.30, .46] | .62 [.54, .70] |
|  | LON | .50 [.43, .57] | .50 [.43, .57] |
|  | NegDep | .49 [.41, .56] | .51 [.44, .59] |
| 18 years | |  |  |
|  | SOC | .42 [.32, .51] | .58 [.49, .68] |
|  | LON | .37 [.27, .47] | .63 [.53, .73] |
|  | NegDep | .54 [.45, .61] | .46 [.39, .55] |

Note. 95% CI in brackets. SOC = sense of coherence; LON = loneliness; NegDep = negative dependent life events.

**Table S5**

*Standardized Parameter Estimates from the Bivariate Cholesky Decomposition Models*

| Model | | A_11_ | A_12_ | A_22_ | E_11_ | E_12_ | E_22_ |
| --- | --- | --- | --- | --- | --- | --- | --- |
| SOC-LON and BPD traits ^a^ | |  |  |  |  |  |  |
|  | 12–13 years | .68 [.60, .75] | .36 [.20, .50] | .61 [.50, .69] | .73 [.66, .80] | .05 [-.05, .15] | .70 [.66, .74] |
|  | 14–15 years | .74 [.68, .78] | .33 [.23, .42] | .63 [.56, .68] | .67 [.62, .73] | .15 [.07, .22] | .69 [.65, .73] |
|  | 16–17 years | .70 [.64, .75] | .47 [.38, .55] | .54 [.45, .61] | .72 [.67, .77] | .10 [.02, .17] | .70 [.66, .73] |
|  | 18 years | .65 [.57, .72] | .50 [.40, .59] | .51 [.39, .59] | .76 [.70, .82] | .15 [.07, .23] | .69 [.65, .73] |
| SOC-LON-NegDep and BPD traits ^b^ | |  |  |  |  |  |  |
|  | 12–13 years | .78 [.71, .82] | .39 [.26, .51] | .59 [.49, .67] | .63 [.57, .70] | .06 [-.05, .16] | .70 [.66, .74] |
|  | 14–15 years | .77 [.73, .81] | .36 [.26, .44] | .61 [.54, .67] | .63 [.58, .69] | .16 [.08, .24] | .69 [.65, .73] |
|  | 16–17 years | .74 [.69, .78] | .51 [.43, .58] | .50 [.41, .57] | .67 [.62, .72] | .10 [.03, .17] | .70 [.66, .73] |
|  | 18 years | .69 [.62, .75] | .50 [.41, .59] | .50 [.40, .58] | .73 [.66, .79] | .16 [.08, .24] | .69 [.65, .73] |

*Note.* 95% CI in brackets. ^a^ Variable 1: the predicted scores for BPD traits derived from linear regression analyses with SOC and LON at different ages as independent variables. Variable 2: BPD traits. ^b^ Variable 1: the predicted scores for BPD traits derived from linear regression analyses with SOC, LON and NegDep at different ages as independent variables. Variable 2: BPD traits. A = additive genetic influences; E = non-shared environmental influences; _11_ = Genetic and environmental influences on the predicted scores; _12_ = Genetic and environmental influences on the predicted scores, contributing to variance in BPD traits; _22_ = Genetic and environmental influences unique to BPD traits.

**Table S6**

*Genetic and Environmental Correlations Derived from the Bivariate Cholesky Decomposition Models*

|  | |  | Non-shared environmental correlation with BPD traits [95% CI] |
| --- | --- | --- | --- |
|  | | Genetic correlation with BPD traits [95% CI] |  |
| Variable ^a^ | |  |  |
| SOC and LON | |  |  |
|  | 12–13 years | .50 [.29, .70] | .08 [-.07, .22] |
|  | 14–15 years | .46 [.32, .60] | .21 [.10, .32] |
|  | 16–17 years | .65 [.54, .77] | .14 [.03, .24] |
|  | 18 years | .70 [.57, .83] | .21 [.10, .32] |
| SOC, LON and NegDep | |  |  |
|  | 12–13 years | .55 [.37, .72] | .08 [-.07, .23] |
|  | 14–15 years | .50 [.37, .62] | .23 [.12, .34] |
|  | 16–17 years | .71 [.61, .81] | .14 [.04, .24] |
|  | 18 years | .71 [.59, .82] | .23 [.12, .34] |

*Note.* BPD traits = borderline personality disorder traits; SOC = sense of coherence; LON = loneliness; NegDep = negative dependent life events. ^a^ Predicted scores for BPD traits derived from linear regression analyses, with SOC and LON (and SOC, LON and NegDep) at different ages as independent variables.

**Table S7**

*Factor Loadings*

| Variable | Factor |
| --- | --- |
| BPD traits | 0.47 |
| SOC 12–13 years | -0.45 |
| LON 12–13 years | 0.48 |
| SOC 14–15 years | -0.58 |
| LON 14–15 years | 0.64 |
| SOC 16–17 years | -0.47 |
| LON 16–17 years | 0.69 |
| SOC 18 years | -0.66 |
| LON 18 years | 0.62 |

*Note.* BPD traits = borderline personality disorder traits; SOC = sense of coherence; LON = loneliness. **Figure S1**

*Genetic and Environmental Influences on the Association between the Predicted Scores and BPD Traits*


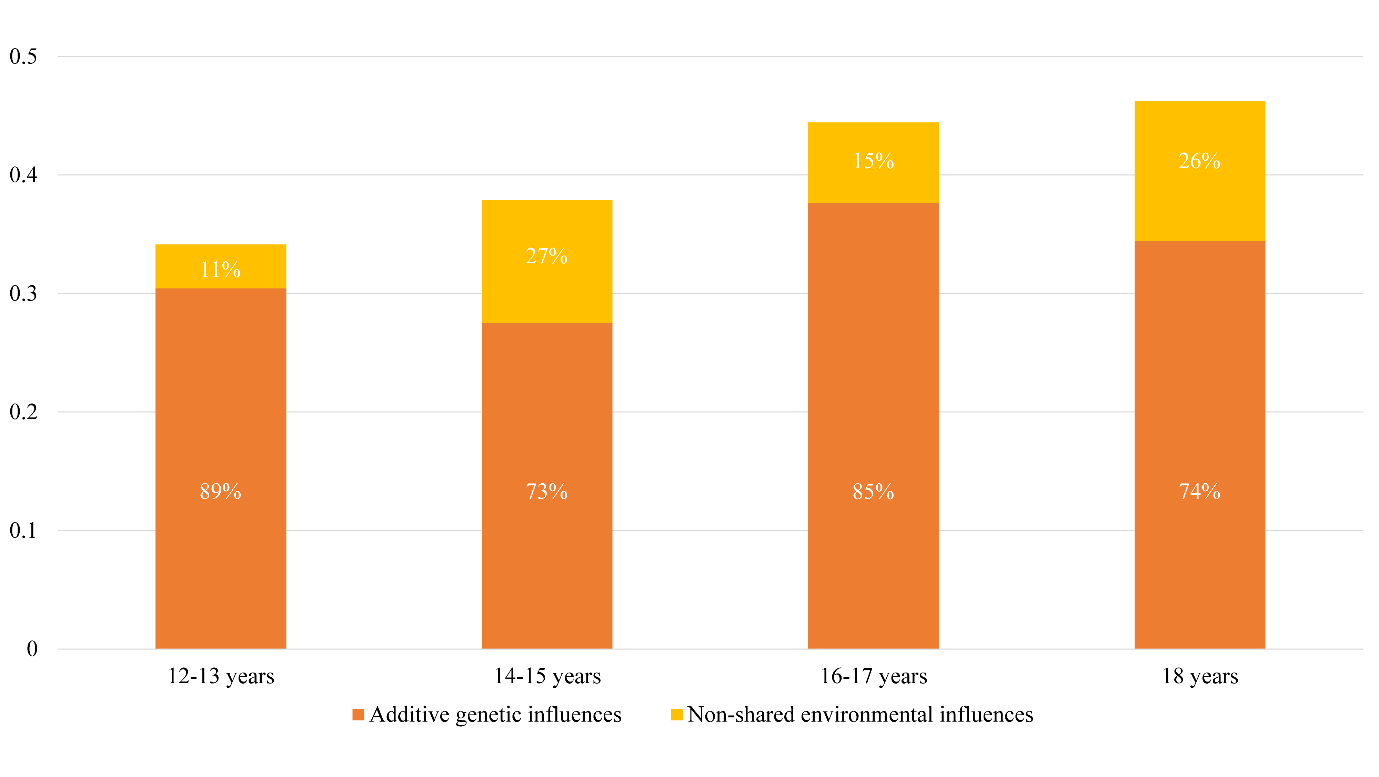


*Note.* BPD traits = borderline personality disorder traits; Predicted scores = predicted scores for BPD traits derived from linear regression analyses, with sense of coherence, loneliness, and negative dependent life events at different ages as independent variables. The height of the bars represents the phenotypic correlation between the predicted scores and BPD traits. The percentages represent the proportions of the phenotypic correlations due to genetic and environmental influences.
